# Editorial: Clinical validation of digital health technologies for personalized medicine

**DOI:** 10.3389/fdgth.2022.831517

**Published:** 2023-04-27

**Authors:** Michelle Khine

**Affiliations:** Department of Biomedical Engineering, Samueli School of Engineering, University of California, Irvine, Irvine, CA, United States

**Keywords:** artificial intelligence, machine learning, clinical trial, intervention, diagnostics, personalized medicine, digital health, health technologies

**Editorial on the Research Topic** Clinical validation of digital health technologies for personalized medicine

If a bright side to this horrific pandemic could be identified, it must be as the requisite catalyst to finally usher in the much-anticipated era of digital health. We are now finally starting to see widespread adoption of digital technologies by healthcare systems. While digital solutions have long transformed every other industry—from retail to transportation to even banking—healthcare has remained the lone, risk-adverse laggard. While its potential and promise are self-evident, and the parallels to other industries obvious, lack of demonstrated clinical benefits, interoperability challenges, and reimbursement issues have consistently plagued digital health from large-scale integration into healthcare. Despite the myriad of health apps literally at our fingertips, and countless wearables that have come and gone, the overall proven benefits have been disappointing. Over the years, this over-hype and under-delivery has resulted in a lot of disillusionment and skepticism.

The old adage “desperate times call for desperate measures” worked to digital health's benefit. Indeed, we as a world would be hard pressed to find a time in recent history more desperate than 2020. So digital health finally got its shot to shine. Forced with not being able to safely see patients in person, the COVID era signaled a more than 4,000% increase in tele-health utilization, in a matter of months. After decades of slow adoption, we are seeing an embrace of digital health solutions by policymakers, healthcare providers, and insurers. Wearable technologies can be used to monitor and warn of COVID infection, and digital passports has proven itself as a way for society to re-open. In the US, the HHS, FDA, and the FCC are investing heavily to solve issues of interoperability, regulations, and funding for virtual care. In the private sector, we saw record funding of companies in the digital health space.

At its core, however, the most critical underpinning to digital health's success and adoption is simply that the technology must work for the end-user. So on our end, we as researchers need to continue to develop robust clinical validation methodologies and studies to objectively determine the value and fit of these emerging technologies. Moreover, we need to reflect on the shortcomings of technologies to date, and how we can improve upon them for next-gen versions that can provide better accuracy, usability, and utility. This is the focus of this Research Topic. To highlight the breadths that digital health technologies span, we have included example technologies from apps to AI to sensors, with papers from around the world. We aim to showcase platforms with clinical study innovation, ways to sustain user/engagement, and roadmaps to demonstrate efficacy.

In *Real-Time Assessment of Stress and Stress Response Using Digital Phenotyping: A Study Protocol,* Eggers et al. presents a trial design to develop a digital phenotype for stress /stress reactions for patients with psychiatric disorders as well as healthy individuals. Specifically, they seek to establish a relationship between the physiological parameters measured by commercially available wearable devices and changes in cortisol levels obtained during everyday stressful situations and a controlled stress situation in both these populations. While there are many apps and wearable devices to monitor psychological well-being and stress, only a tiny fraction of these technologies have has been validated adequately in controlled studies. Studies are often skewed with under-representation of users with psychiatric disorders. For truly effective and personalized technology, fitting to the individual's stress and stress reactions in everyday situations as well as in a controlled laboratory setting is essential.

In *Second-Generation Digital Health Platforms: Placing the Patient at the Center and Focusing on Clinical Outcomes* by Y. Ilan, we explore the barriers that have stymied medical AI's ability to achieve significant, reliable clinical impacts. After a review of first-generation AI in clinical practice and its shortcomings, a second-generation platform is introduced, with a closed-loop dynamic feedback system designed to be responsive to the effect of therapy on clinical outcomes. Instead of relying on large databases, this personalized *n* = 1 tracking builds on the patient longitudinally, and as such can quantify variability patterns in the patient to provide customized therapeutic regimens. Such a dynamic system enables chronic therapies to have sustainable effects, overcoming compensatory mechanisms associated with disease progression and drug resistance.

The final two papers of this Research Topic are focused on sensors. In *Telemonitoring Techniques for Lung Volume Measurement: Accuracy, Artifacts and Effort* by Mannée et al., we explore technologies used for telehealth monitoring of lung function. Such technologies are particularly critical in this pandemic, but adoption has been stymied due to inconclusive effectiveness. This review paper explores several main technologies to track lung volume measurements, including portable spirometry/breath-by-breath analyzers, respiratory inductance and magnetic plethysmography and electrical impedance tomography. The paper critically examines their accuracy, usability, and appropriateness for telehealth applications.

Finally, in *Clinical Validation of a Soft Wireless Continuous Blood Pressure Sensor During Surgery* by Chou et al., we explore a new technology for beat to beat blood pressure monitoring. Whereas the blood pressure cuff is to most common way to monitor blood pressure, it only provides static, time-averaged values and is notorious for being inaccurate. While the invasive arterial line can measure real time beat to beat blood pressure, its invasive nature limits its use to high-risk surgeries and intensive care situations. This primary paper evaluates a soft sensor based on applanation tonometry by comparing it head to head in a surgical setting against the gold-standard arterial line. Importantly, this paper reports results across a wide patient population of ages and body habitus. Such real-world clinical studies against gold standards are critical to proving efficacy as well as utility for new technologies. While much work remains to develop this technology, as is true for all the technologies highlighted in this Research Topic, these papers lay the groundwork and framework to design, rigorously test, critically assess, and improve on emerging technologies.

As we enter the post-COVID age, where digital health technology integration into healthcare will become common-place and second nature, we need to have confidence that the technologies we are using have been rigorously designed, tested, and clinically validated to provide personalized, accurate, and meaningful information. The papers in this Research Topic aim to highlight some recent work as examples of how to get us there.

On a personal note, I urge you to join us in this long overdue revolution in medicine. My beloved mother and best friend suffered from Alzheimer's disease for over a decade and died suddenly last month. Her internist called me the morning after she died to tell me her blood work came back—and everything looks fine. I had taken her for a check-up a week before she died, which was an exercise in futility: the doctor re-explained he could do nothing for the disease that was ravaging her brain and her personality—but otherwise she seemed very strong and healthy. For a decade I had to listen to that inanity. None of it is true and none of it should be acceptable. Medicine failed my mother. We need to do better. This is a call to arms.

Conventional medical sampling is a crude snapshot in time: a single blood draw once a year, a blood pressure measurement at the doctor's office, a urine analysis when a patient complains of symptoms. *Subtle physiological changes that precede clinical deterioration (catastrophic failure in the case of my mom) are all together obscured the by the inherent under-sampling and limited sensitivity of current instruments.* In medicine, an *n* = 1 has conventionally been considered insignificant. But I would argue it is so only because of the sparsity of data that medicine has collected on each patient. If we continuously tracked and monitored data (much as is standard with any recent car), we would have a wealth of data per patient. We could then understand effects of diet, exercise, genetics, environment, lifestyle choices, and interventions as the patient would serve as his/her own control. Every patient is significant—and it is time medicine started treating them that way.

